# A novel senescence-related lncRNA signature that predicts prognosis and the tumor microenvironment in patients with lung adenocarcinoma

**DOI:** 10.3389/fgene.2022.951311

**Published:** 2022-11-03

**Authors:** Xueying Fang, Enmin Huang, Xiaopeng Xie, Kai Yang, Shuqian Wang, Xiaoqing Huang, Mei Song

**Affiliations:** ^1^ Department of Respiratory and Critical Care Medicine, The Second Affiliated Hospital of Shantou University Medical College, Shantou, China; ^2^ Guangdong Provincial Key Laboratory of Colorectal and Pelvic Floor Diseases, Supported by National Key Clinical Discipline, Department of Gastroenterological Surgery and Hernia Center, The Sixth Affiliated Hospital, Sun Yat-sen University, Guangzhou, China

**Keywords:** cellular senescence, lncRNA, prognosis, tumor microenvironment, immunotherapy, lung adenocarcinoma

## Abstract

**Background:** Cellular senescence has recently been considered a new cancer hallmark. However, the factors regulating cellular senescence have not been well characterized. The aim of this study is to identify long non-coding RNAs (lncRNAs) associated with senescence and prognosis in patients with lung adenocarcinoma (LUAD).

**Methods:** Using RNA sequence data from the Cancer Genome Atlas Lung Adenocarcinoma (TCGA-LUAD) and senescence genes from the CellAge database, a subset of senescence-related lncRNAs was first identified. Then, using univariate and multivariate Cox regression analyses, a senescence lncRNA signature (LUADSenLncSig) associated with LUAD prognosis was developed. Based on the median LUADSenLncSig risk score, LUAD patients were divided into high-risk and low-risk groups. Kaplan-Meier analysis was used to compare the overall survival (OS) in the high- and low-risk score subgroups. Differences in Gene Set Enrichment Analysis (GSEA), immune infiltration, tumor mutation burden (TMB), tumor immune dysfunction and exclusion (TIDE) module score, chemotherapy, and targeted therapy selection were also compared between the high-risk and low-risk groups.

**Results:** A prognostic risk model was obtained consisting of the following nine senescence-related lncRNAs: LINC01116, AC005838.2, SH3PXD2A-AS1, VIMS-AS1, SH3BP5-AS1, AC092279.1, AC026355.1, AC027020.2, and LINC00996. The LUADSenLncSig high-risk group was associated with poor OS (hazard ratio = 1.17, 95% confidence interval = 1.102–1.242; *p* < 0.001). The accuracy of the model was further supported based on receiver operating characteristic (ROC), principal component analysis (PCA), and internal validation cohorts. In addition, a nomogram was developed consisting of LUADSenLncSig for LUAD prognosis, which is consistent with the actual probability of OS. Furthermore, immune infiltration analysis showed the low-risk group had a stronger anti-tumor immune response in the tumor microenvironment. Notably, the levels of immune checkpoint genes such as CTLA-4, PDCD-1, and CD274, and the TIDE scores were significantly higher in the low-risk subgroups than in high-risk subgroups (*p* < 0.001). This finding indicates the LUADSenLncSig can potentially predict immunotherapy efficacy.

**Conclusion:** In this study, a lncRNA signature, LUADSenLncSig, that has dual functions of senescence phenotype identification and prognostic prediction as well as the potential to predict the LUAD response to immunotherapy was developed.

## Introduction

Lung cancer is a common malignant tumor, with the second highest incidence and highest mortality rate in the world (Ferlay et al., 2021). More than 1.3 million people die from lung cancer worldwide each year (Siegel et al., 2021). The 5-year survival rate for non-small cell lung cancer (NSCLC) is 23%, however, for small cell lung cancer is approximately only 6% (Siegel et al., 2021). Lung adenocarcinoma (LUAD) is the most common type of NSCLC, accounting for roughly 40% of all cases (Seguin et al., 2022). LUAD is more difficult to prevent and treat than lung squamous cell carcinoma, which is mostly associated with smoking (Seguin et al., 2022). Despite recent advances in genomics and targeted therapies for lung cancer, the disease is susceptible to drug resistance, resulting in a poor prognosis. In recent years, the development of immunotherapy has led to a new treatment direction for lung cancer. In 2015, CheckMate-063, a phase II single-arm trial, was the first large study in which the efficacy of immunotherapy in NSCLC was reported (Rizvi et al., 2015). In two phase III studies, CheckMate-017 (Brahmer et al., 2015) and CheckMate-057 (Borghaei et al., 2015), patients with NSCLC treated with nivolumab or docetaxel had significantly better overall survival (OS), response rate, and progression-free survival (PFS) when treated with nivolumab than docetaxel. Due to these impressive results, the FDA approved nivolumab as a second-line treatment for NSCLC in March 2015. Unfortunately, most LUAD patients do not respond to immune checkpoint inhibitor (ICI) therapies due to a lack of appropriate patient-selective biomarkers. To improve patient outcomes, new effective immunotherapy markers need to be developed.

Cellular senescence is a program of stable cell cycle arrest in response to various intrinsic and extrinsic stimuli to remove senescent cells to maintain body homeostasis (Kumari and Jat, 2021). Cellular senescence is primarily caused by progressive telomere shortening, telomere structure change, mitosis, carcinogenic activation, ionizing radiation, oxidation, genotoxic stress, epigenetic changes, chromatin disorder, protein steady-state disorder, mitochondrial dysfunction, inflammation, tissue damage signal, radiation therapy, or chemotherapy (Pazolli et al., 2012; García-Prat et al., 2016; Mikuła-Pietrasik et al., 2020; d'Adda di Fagagna et al., 2003; Passos et al., 2010). Both cancer and senescence are caused by the accumulation of cell damage. Previous research has shown that senescence is both beneficial and detrimental in the process of tumorigenesis and development, and viewed as an example of the dichotomy of multiple effects in the evolutionary process (Ohtani et al., 2012; Schosserer et al., 2017). Conversely, senescence causes senescent cells to enter a permanent cell stagnation cycle to maintain tissue homeostasis and prevent tumor formation (Wang et al., 2020a). However, when senescent cells are not cleared by the immune system and accumulate, cell senescence may have harmful results, promoting the occurrence, development, invasion, and metastasis of tumors through multiple pathways (Wang et al., 2020a).

Phenotypic changes during cell senescence are controlled by changes in the specific proteins expressed. These processes are mainly regulated by proteins linking DNA and RNA as well as various non-coding RNAs, including long non-coding RNAs (lncRNAs) (Grammatikakis et al., 2014). LncRNA-XIST expression was shown downregulated in senescent cells and inhibited NSCLC cell proliferation and promoted apoptosis by triggering cell necrosis mediated by the miR-335/SOD2/ROS signaling pathway, thereby inhibiting NSCLC progression (Liu et al., 2019). LncRNA H19 regulates the imprinting of gene clusters containing H19 and insulin-like growth factor 2 (IGF2) (Monnier et al., 2013). Both IGF2 (Issa et al., 1996) and H19 (Gabory et al., 2010) are associated with growth, proliferation, cell circumference, apoptosis, and senescence. LncRNA H19 is also highly expressed in lung cancer, and by inhibiting the function of miR-200a, upregulates the expression of ZEB1 and ZEB2, promoting the epithelial-mesenchymal transition and enhancing lung cancer cell proliferation and metastasis (Zhao et al., 2019). Taken together, lncRNAs can characterize cellular senescence and serve as an important tool in determining LUAD patient prognosis.

In the present study, a senescence-related lncRNA signature was constructed and shown to predict the prognosis of LUAD patients. In addition, a nomogram was developed that included a LUAD senescence-related lncRNA signature (LUADSenLncSig). Clinical factors, gene enrichment, mutations, immune cell infiltration, and potential response to targeted therapy and immunotherapy were further compared in the LUADSenLncSig high-risk and low-risk groups. In the present study, the regulatory network of cellular senescence was investigated and hypothesized to improve the efficacy of individualized treatment for LUAD.

## Materials and methods

### Dataset and sample extraction

The LUAD RNA sequencing data, clinical, and mutations were downloaded from The Cancer Genome Atlas Lung Adenocarcinoma (TCGA-LUAD) database (https://portal.gdc.cancer.gov/). Data from 539 LUAD patients were initially collected. Patients with incomplete follow-up information, survival <30 days, or lack of complete clinicopathological data were excluded from the follow-up analysis and finally, 448 patients were retained. The online database CellAge (http://genomics.senescence.info/cells) was used to download 279 senescence-related genes identified from gene manipulation experiments (Avelar et al., 2020).

### Differentially expressed genes (DEGs) of cellular senescence in LUAD and normal tissue

The threshold of log_2_ fold change absolute value >1 and a false detection rate (FDR) < 0.05 were used to detect the differentially expressed genes (DEGs) between 539 tumor tissues and 59 normal tissues. The R package limma was used to visualize DEGs. Enrichment analysis of DEGs was performed using the Kyoto Encyclopedia of Gene and Genome (KEGG) and Gene Ontology (GO).

### Identification of a lncRNAs senescence-related signature (LUADSenLncSig) associated with LUAD prognosis

The senescence-related lncRNA-mRNA co-expression gene expression network was developed using Pearson correlation coefficient absolute value >0.3 and *p* < 0.001 as thresholds to identify senescence-related lncRNAs. The Sankey diagram generated using the R package ggalluvial and Cytoscape software (version 3.7.2) were used to visualize the lncRNA-mRNA co-expression network. First, univariate Cox regression analysis was used to identify lncRNAs associated with LUAD prognosis, then the lncRNAs were further incorporated into multivariate Cox regression analysis to construct the prognosis model of LUAD; this lncRNA senescence-related signature was termed LUADSenLncSig. The risk score of the prognostic model was calculated using the following formula and LUAD patients were divided into high- and low-risk groups based on the median risk score: risk score = explncRNA1 × coef lncRNA1 + explncRNA2 × coef lncRNA2 + explncRNAi × coef lncRNAi. To determine whether the LUADSenLncSig risk score was an independent prognostic index, the clinicopathological variables were included in univariate and multivariate Cox regression analyses. To investigate the distribution of living conditions, the risk score level was used. Furthermore, the accuracy of the risk model was assessed using the receiver operating characteristic (ROC) curve. The R package pheatmap was used to display the clinicopathological variables of the high- and low-risk groups. The distribution of patients with different risk scores was assessed using principal component analysis (PCA) and the visualization was performed using R package scatterplot3d.

### Construction of the nomogram

A nomogram was constructed by combining risk scores and clinicopathological features and using the R package rms to predict survival at 1, 3, and 5 years in patients with LUAD. Calibration curves were used to determine if the predicted survival matched the actual survival.

### Gene Set Enrichment Analysis (GSEA) of the senescence-related lncRNA predictive signature

Gene Set Enrichment Analysis (GSEA) was performed for the high- and low-risk groups to identify different functional enrichment of the two groups. Significant biological processes and pathways were enriched at the threshold of nominal (NOM) *p* < 0.05 and the FDR q-value < 0.25. The R package ggplot2 was used to visualize the results.

### Analysis of somatic mutations and tumor mutation burden (TMB)

The number of somatic non-synonymous mutations in each sample was calculated using the R package maftools (Mayakonda et al., 2018). The tumor mutation burden (TMB) uses non-synonymous and code-shifting indels and a 5% detection limit to calculate the number of somatic, coding, base replacement, and insert-deletion mutations found in the genome database per megabase. The TMB was also compared between the high-risk and low-risk groups and survival curves and risk scores for TMB were plotted.

### Estimation of immune infiltration

The immune cell infiltration fraction and immune-related pathway activity in high- and low-risk LUAD samples were assessed using single-sample GSEA (ssGSEA) (Rooney et al., 2015) and the CIBERSORT algorithm as cross-validation (Newman et al., 2015). The Wilcoxon rank-sum test was used to determine whether a significant difference existed in immune cell proportions between the low-risk and high-risk groups.

### Potential relationship between the LUADSenLncSig and immunotherapy, chemotherapy, and target therapy

First, the differential expression of 40 immune checkpoints in high-risk and low-risk groups was compared. Furthermore, the tumor immune dysfunction and exclusion (TIDE, http://tide.dfci.harvard.edu/) module was used to distinguish potential immunotherapy responses between high-risk and low-risk groups. This module predicts anti-PD1 and anti-CTLA4 treatment responses based on the transcriptional expression profiles of patient genomes before treatment. In addition, to further evaluate the role of LUADSenLncSig in predicting the therapeutic response of LUAD, the half-maximal inhibitory concentration (IC_50_) of commonly used chemotherapeutic drugs and targeted therapeutic drugs was calculated. Wilcoxon signed-rank test and R package pRRophetic were used to compare and visualize the IC_50_ values in the high-risk and low-risk groups.

### Statistical analysis

The Wilcoxon test was used to compare the expression of senescence-related DEGs in cancer tissues and normal tissues. The Kaplan-Meier method and log-rank test were used to compare the OS rate between the high-risk and low-risk groups. The survivalROC package was used to generate ROC curves and calculate the area under the curve (AUC). The Kruskal–Wallis test was used to compare the differences between groups. The clinical data were analyzed using the chi-square test or the Fisher’s exact test. A Pearson correlation coefficient was used to evaluate the relationship between lncRNA expression and immune infiltration and immune checkpoint gene expression. All statistical analyses were performed using R software (version 4.1.2).

## Results

### Enrichment analysis of senescence-related DEGs in LUAD

**FIGURE 1:**
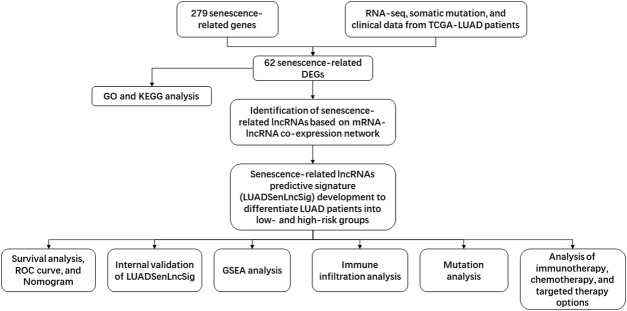
Flowchart of the present study. TCGA, The Cancer Genome Atlas; RNA-seq, RNA sequence; LUAD, lung adenocarcinoma; DEGs, differentially-expressed genes; GO, Gene Ontology; KEGG, Kyoto Encyclopedia of Genes and Genomes; lncRNAs, long non-coding RNAs; ROC, receiver operating characteristic; GSEA, Gene Set Enrichment Analysis.

**FIGURE 2:**
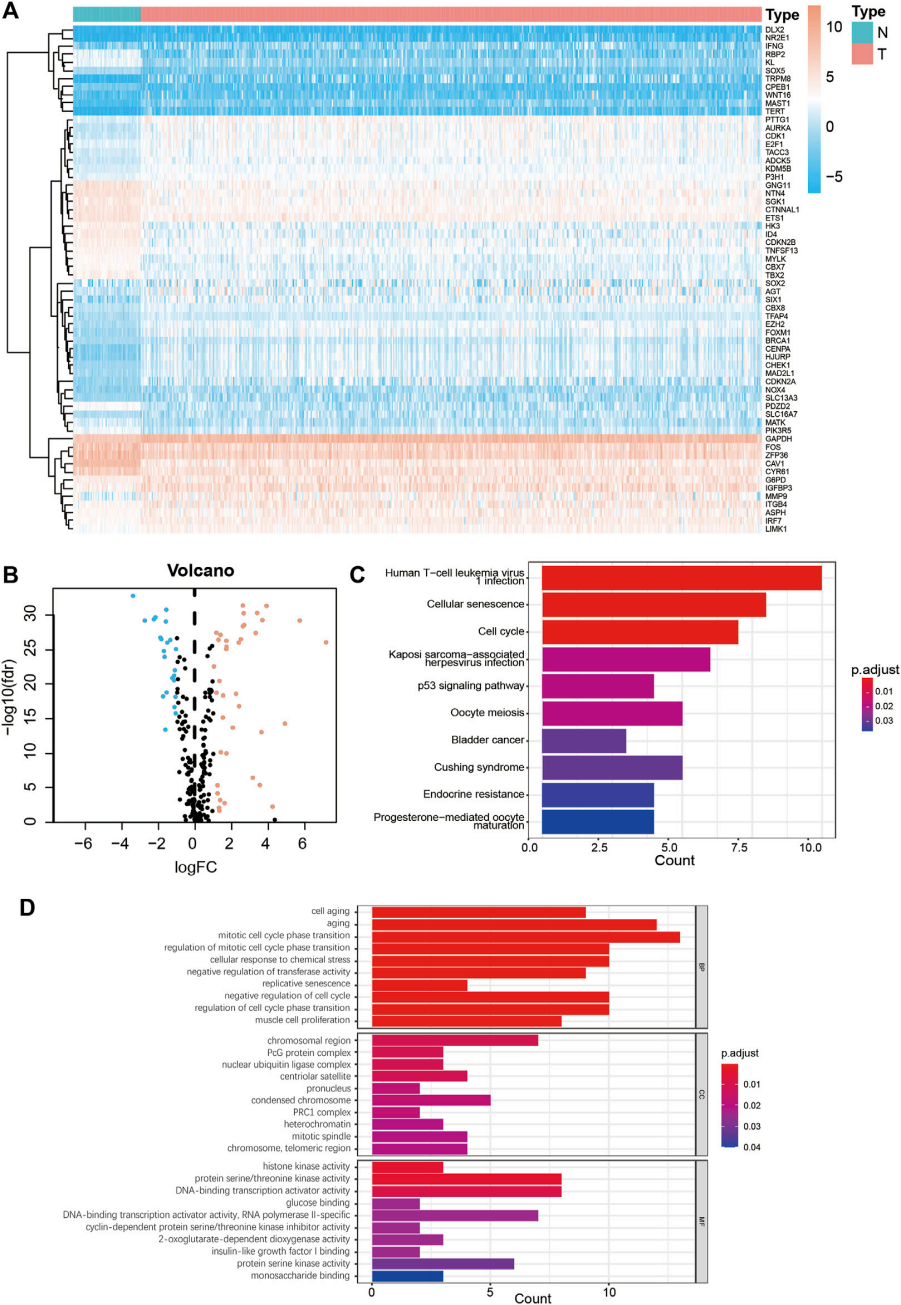
Enrichment analysis of senescence DEGs in LUAD. **(A)** Heatmap of 62 senescence DEGs in normal and LUAD tumor tissues. **(B)** Volcano plot of 279 senescence genes in normal and LUAD tumor tissues. Pink dots represent genes that have been upregulated in tumor tissues and blue dots represent genes that have been downregulated in tumor tissues. **(C)** KEGG analysis of senescence DEGs. **(D)** GO analysis of senescence DEGs. DEGs, differentially expressed genes; LUAD**,** lung adenocarcinoma; N, normal tissues; T, tumor tissues; FC, fold change; KEGG, Kyoto Encyclopedia of Genes and Genomes; GO, Gene Ontology; FDR, false detection rate; BP, biological process; CC, cellular components; MF, molecular function.

The study flow chart is shown in Figure 1. First, the expression levels of 279 senescence-related genes in tumor and normal tissues were compared to determine if the genes were abnormally expressed in tumor tissues (Supplementary Table S1). In LUAD tumor tissues, 23 of 62 differentially expressed genes were downregulated and 39 were upregulated (Figures 2A,B, Supplementary Table S2). KEGG pathway analysis revealed the five most enriched pathways were human T-cell leukemia virus one infection, cellular senescence, cell cycle, Kaposi sarcoma-associated herpesvirus infection, and p53 signaling pathway (Figure 2C). Conversely, GO analysis showed the five most enriched categories were cell aging, aging, mitotic cell cycle phase transition, regulation of mitotic cell cycle phase transition and cellular response to chemical stress (Figure 2D). These findings indicate the DEGs are primarily involved in cell senescence, cell cycle, virus infection, cell stress, and cell apoptosis.

### Construction of the LUADSenLncSig

**FIGURE 3:**
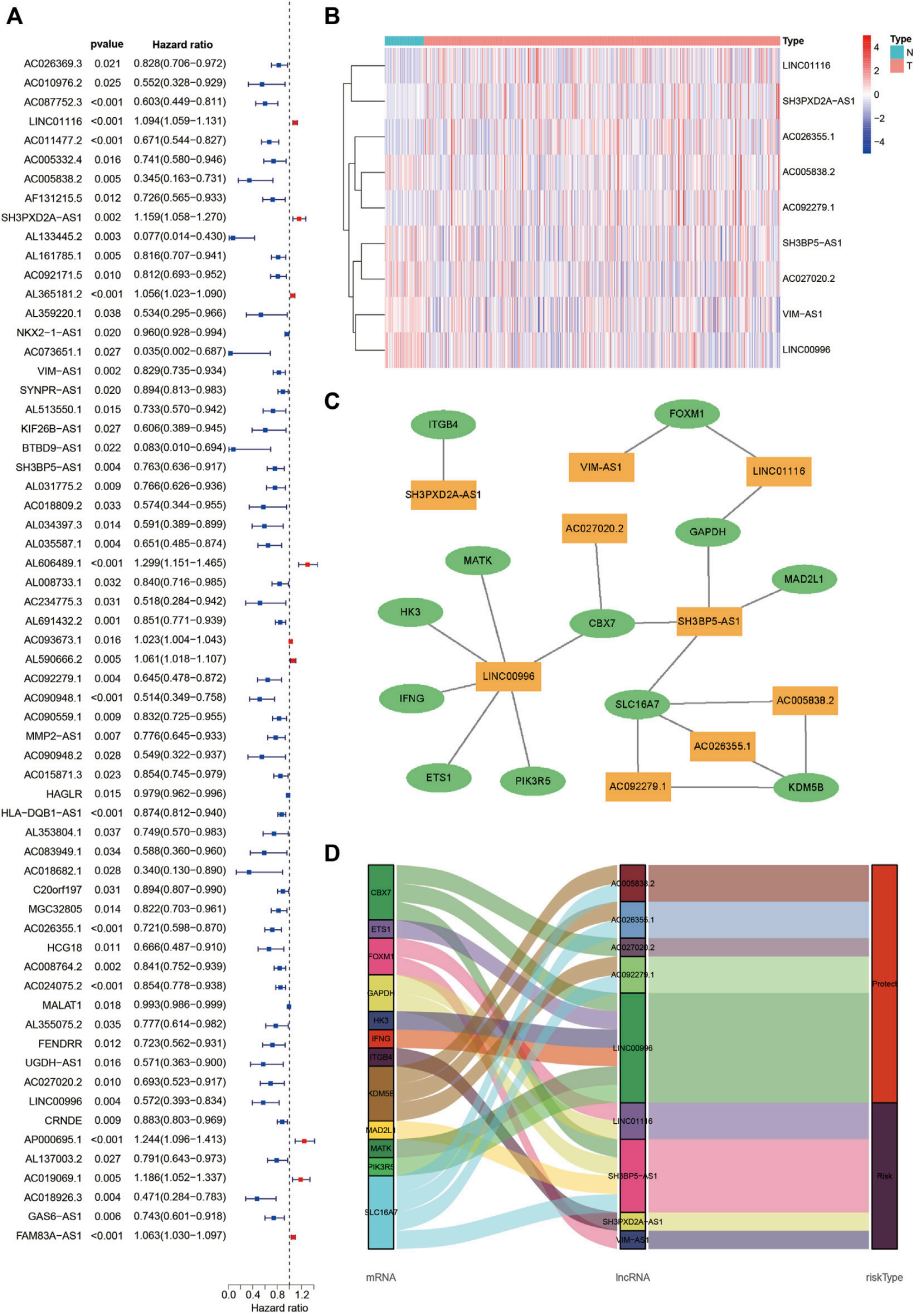
Identification of senescence-related lncRNAs associated with LUAD prognosis and lncRNA-mRNA co-expression network construction. **(A)** The Forest plot shows 62 senescence-related lncRNAs with HRs, 95% CIs, and *p*-values for their associated LUAD prognosis based on univariate Cox proportional hazards analysis. **(B)** A heatmap depicts the expression levels of nine senescence-related lncRNAs identified associated with LUAD prognosis based on multivariate Cox regression analysis. **(C)** The lncRNA-mRNA co-expression network of the senescence-related signature. Yellow squares represent lncRNAs and green ellipses represent mRNA. The expression levels of the nine senescence lncRNAs were associated with the levels of 12 senescence mRNAs. **(D)** The Sankey diagram depicts the relationships between senescence lncRNAs, mRNAs, and risk type. LncRNAs, long non-coding RNAs; HRs, hazard ratios; CIs, confidence intervals; N, normal tissues; T, tumor tissues; LUAD, lung adenocarcinoma.

Pearson correlation analysis identified 1,081 lncRNAs associated with senescence (Supplementary Table S3). Among the lncRNAs, 62 were associated with LUAD prognosis (Figure 3A). In addition, nine senescence-related lncRNAs (LINC01116, AC005838.2, SH3PXD2A-AS1, VIMS-AS1, SH3BP5-AS1, AC092279.1, AC026355.1, AC027020.2, and LINC00996) were screened using multivariate Cox regression analysis to form the LUADSenLncSig prediction signature. Figure 3B depicts a heat map of the LUADSenLncSig lncRNA expression levels in LUAD patients. Cytoscape software was used to visualize the senescence-related lncRNA-mRNA expression network (Figure 3C, |*R* ^2^| > 0.3, *p* < 0.001). AC005838.2, AC026355.1, AC027020.2, AC092279.1 and LINC00996 were protective factors and LINC01116, SH3PXD2A-AS1, VIM-AS1 and SH3BP5-AS1 risk factors based on the Sankey diagram in Figure 3D.

### Correlation between LUADSenLncSig and prognosis of LUAD patients

**FIGURE 4:**
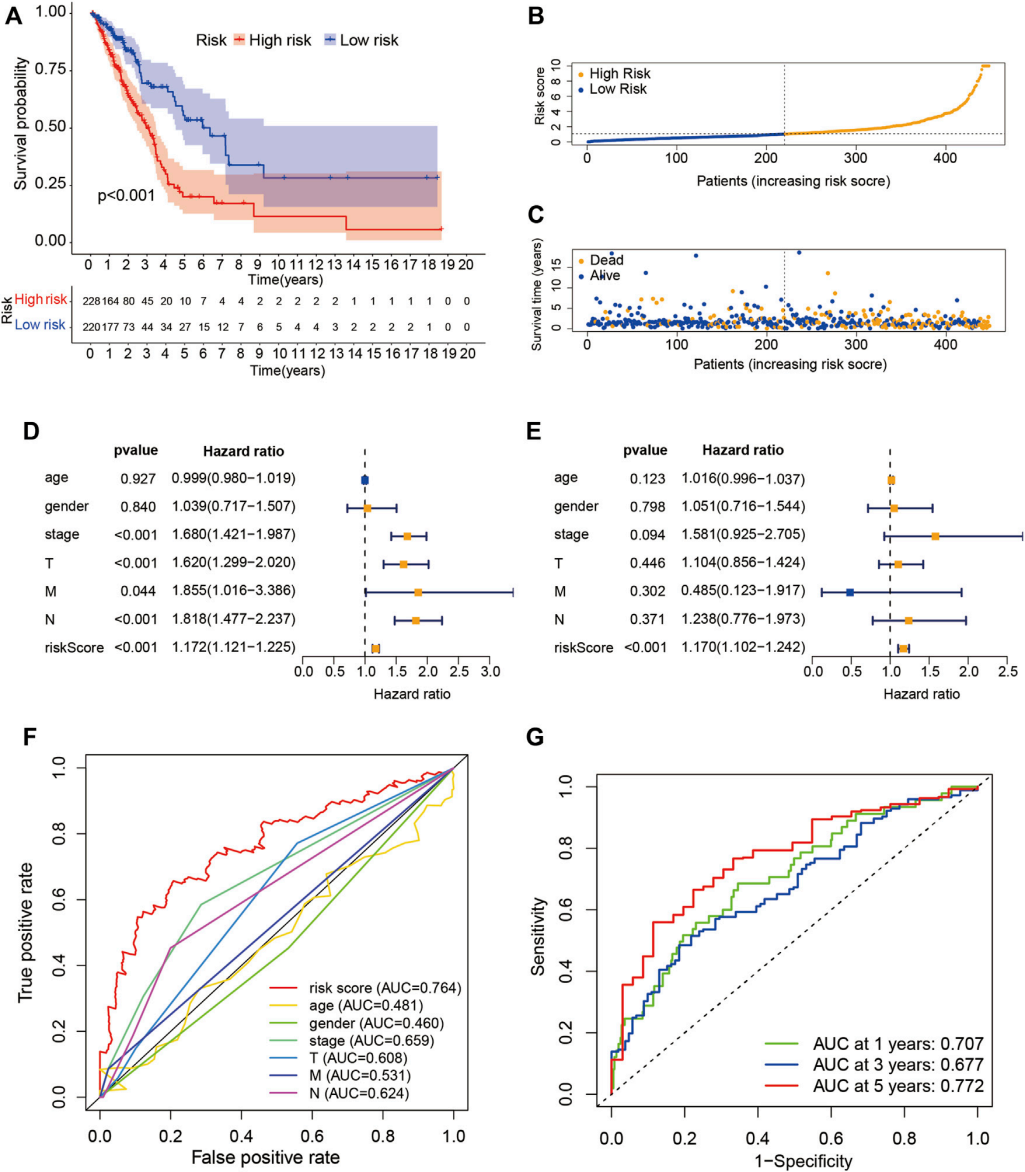
Prognostic value of the risk score determined based on the senescence-related lncRNA (LUADSenLncSig) predictive model. **(A)** Kaplan–Meier curves for OS in the high- and low-risk groups stratified based on the median of risk scores determined using LUADSenLncSig. **(B)** Risk curve based on the risk score for each sample; yellow dot indicates a high risk and blue dot indicates a low risk. **(C)** Scatterplot based on the survival status of each sample. Yellow and blue dots indicate the status of survival or death, respectively. **(D)** Forest plot of the clinicopathological characteristics and LUADSenLncSig risk score for univariate Cox regression analysis and **(E)** multivariate Cox regression analysis. **(F)** The AUC between the risk score and other clinicopathological variables compared using ROC. **(G)** Time-dependent ROC curves for 1-year, 3-year, and 5-year survival for the LUADSenLncSig. OS, overall survival; LUAD, lung adenocarcinoma, lnc, long non-coding; LUADSenLncSig, lung adenocarcinoma senescence lncRNA signature; AUC, area under the curve; ROC, receiver operating characteristic curve; T, tumor; M, distant metastasis; N, lymph node metastasis.

The risk score of LUADSenLncSig was calculated as follows: risk score = (0.348 × LINC01116 expression) + (−0.889 × AC005838.2 expression) + (0.516 × SH3PXD2A-AS1 expression) + (−0.429 × VIM-AS1 expression) + (0.883 × SH3BP5-AS1 expression) + (−0.611 × AC092279.1 expression) + (−0.566 × AC026355.1 expression) + (−0.918 × AC027020.2 expression) + (−0.869 × LINC00996 expression). The formula was used to calculate the risk score of each patient and the patients were divided into two groups based on the median risk score: high-risk group (n = 228) and low-risk group (n = 220) (Figure 4A). Kaplan-Meier analysis showed the OS was significantly shorter in the high-risk group than in the low-risk group (Figure 4A). Figures 4B,C show risk scores and survival statistics for individual patients, with a greater number of deaths with increasing risk scores. Univariate and multifactorial Cox regression analyses showed the LUADSenLncSig risk score was an independent prognostic factor for LUAD (Figures 4D,E) with an AUC of 0.764 and the best predictor of LUAD prognosis compared with other clinicopathological variables (Figure 4F). The AUC of the 1-year, 3-year, and 5-year ROC curves were 0.707, 0.677, and 0.772, respectively, indicating that LUADSenLncSig performed well in LUAD prognosis (Figure 4G).

**FIGURE 5:**
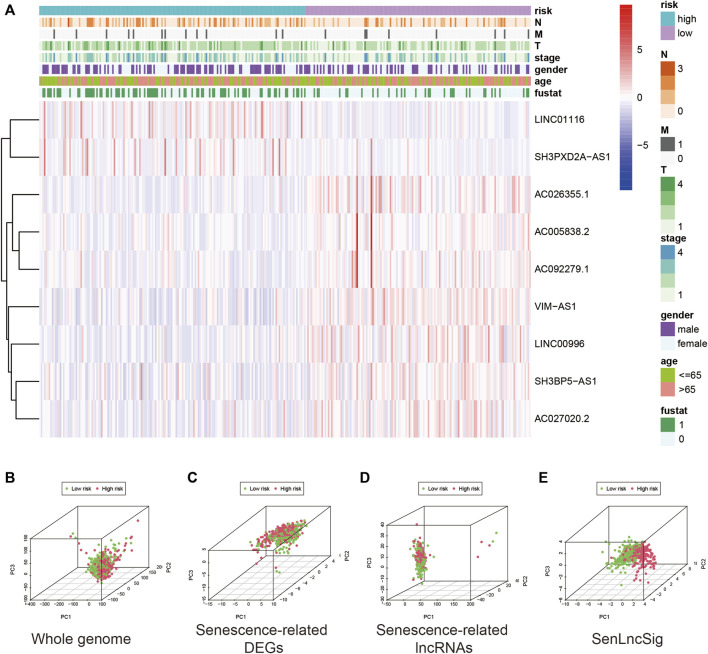
Expression levels of the nine lncRNAs based on clinicopathological variable stratification and PCA of the different gene sets used to classify patients into different risk groups. **(A)** Nine senescence-related lncRNAs and clinicopathological variables were distributed as a heat map for the high- and low-risk groups. PCA based on **(B)** whole-genome mRNA transcripts, **(C)** senescence-related mRNAs **(D)** senescence-related lncRNAs, and **(E)** risk model including the nine LUADSenLncSig senescence-related lncRNAs. Patients with high-risk scores are shown in red and subjects with low-risk scores are shown in green. LncRNAs, long non-coding RNAs; PCA, principal component analysis; LUAD, lung adenocarcinoma; LUADSenLncSig, LUAD senescence lncRNA signature; T, tumor; N, lymph node metastasis; M, distant metastasis.

Figure 5A depicts the expression levels of the nine lncRNAs and clinicopathological factors in the LUADSenLncSig model. To differentiate between high-risk and low-risk patients, PCA with genome-wide, senescence-related DEGs, senescence-related lncRNAs, and LUADSenLncSig, was performed (Figures 5B–E). The LUADSenLncSig model clearly distinguished low-risk and high-risk populations, as shown in Figure 5E, demonstrating the accuracy of the model.

**FIGURE 6:**
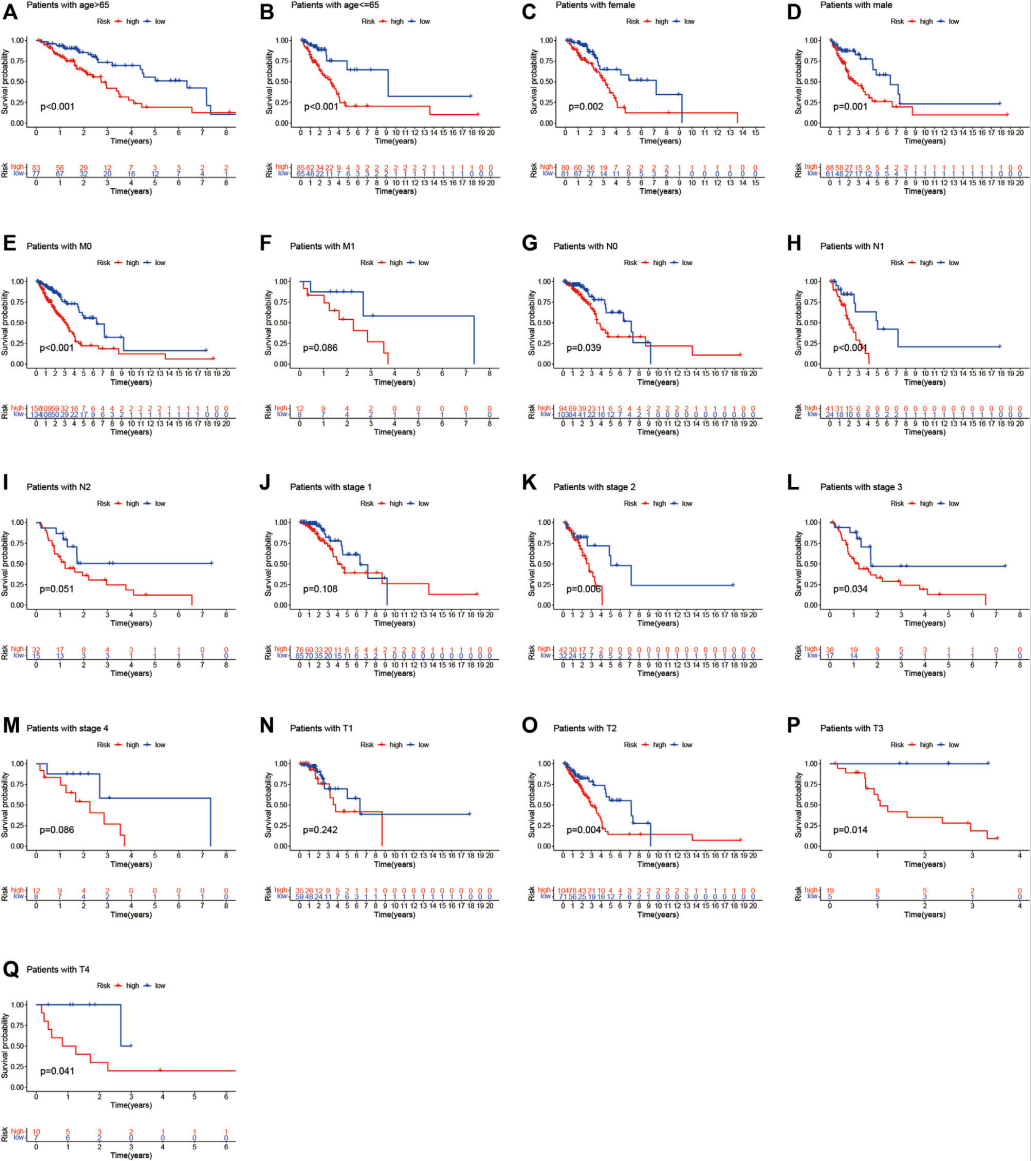
Kaplan–Meier survival curves for low- and high-risk groups classified based on clinicopathological variables. **(A,B)** Age; **(C,D)** Sex; **(E,F)** M stage; **(G–I)** N stage; **(J–M)** Overall stage; **(N–Q)** T stage. T, tumor; N, lymph node metastasis; M, distant metastasis.

Furthermore, Figure 6A–Q shows the relationship between clinical parameters and risk score. Significant correlations were observed between the risk score and age (> 65 years and ≤ 65 years, Figure 6A and B), sex (female and male, Figure 6C,D), M0 stage (Figure 6E), N0 and N1 stage (Figure 6G,H), overall TNM stage 2 and 3 (Figure 6K,L), and T2, T3, and T4 stage (Figures 6O–Q). The LUADSenLncSig risk score was proven an independent prognostic risk factor for patients with LUAD.

### Construction of the nomogram

**FIGURE 7:**
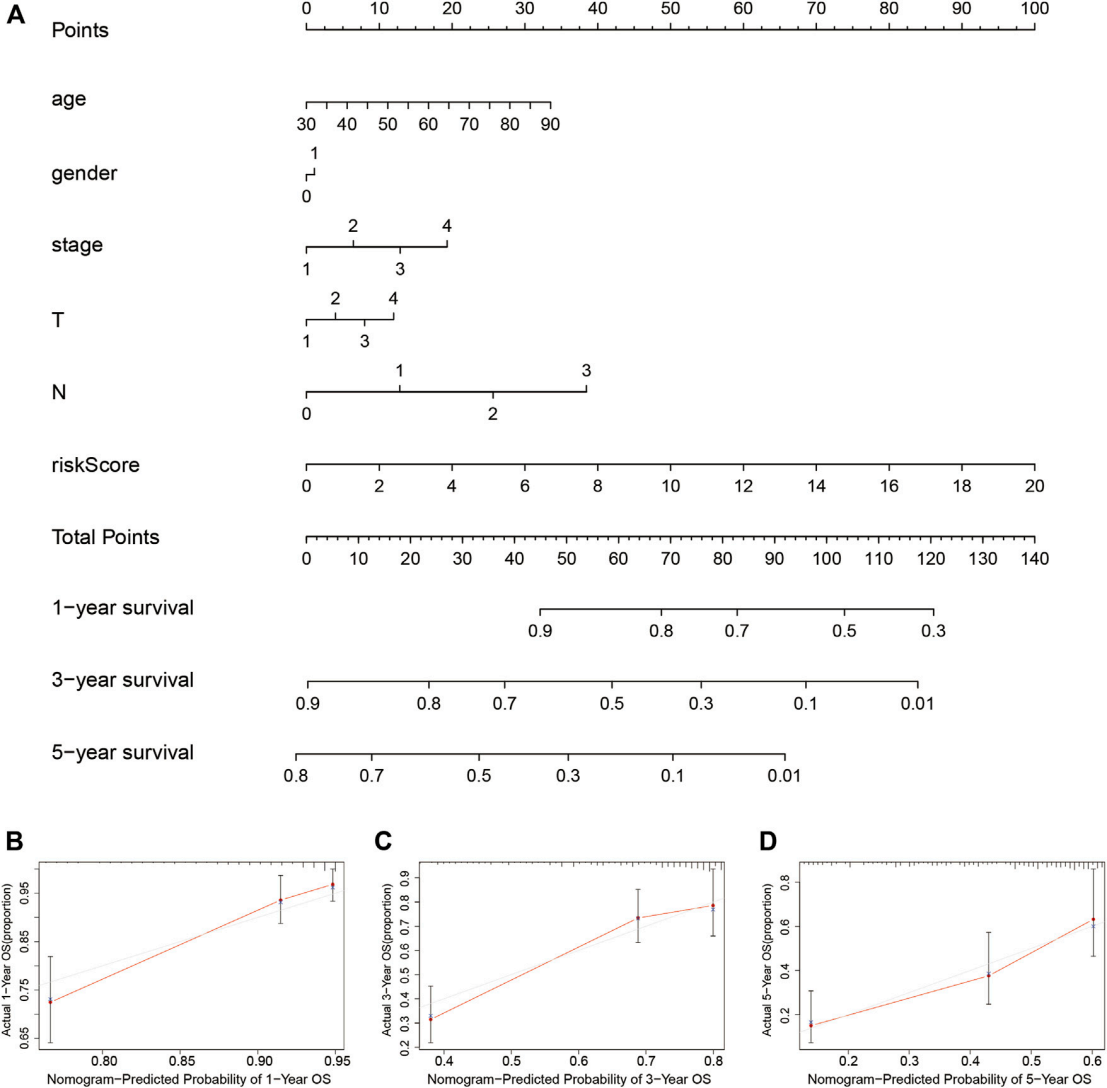
Construction and verification of the nomogram **(A)** The nomogram combining clinicopathological variables and risk scores predicts the 1-year, 3-year, and 5-year survival probability of LUAD patients. The calibration curves evaluate the consistency of predicted and actual OS at **(B)** 1 year **(C)** 3 years, and **(D)** 5 years. LUAD, lung adenocarcinoma; T, tumor; N, lymph node metastasis; M, distant metastasis; OS, overall survival.

A nomogram clinical prognostic assessment plot was established using the LUADSenLncSig risk score and other clinicopathological factors to assess the probability of survival at 1, 3, and 5 years for patients with LUAD (Figure 7A). Based on the three calibration plots, the nomogram-estimated mortality was similar to the actual mortality (Figures 7B–D).

### Internal validation of the LUADSenLncSig

**FIGURE 8:**
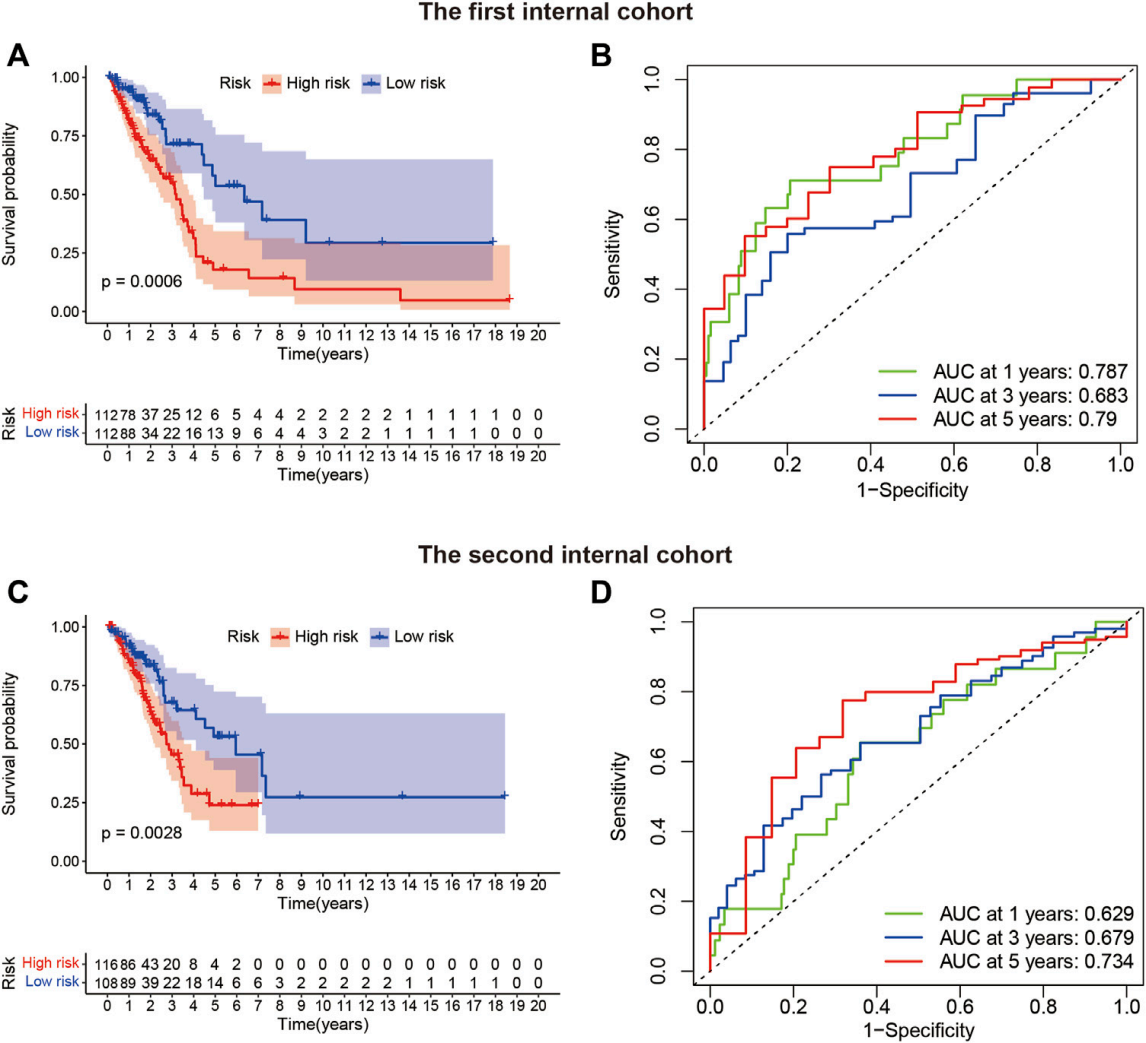
Internal validation of LUADSenLncSig for OS. **(A)** Kaplan–Meier survival curves for the first internal cohort (n = 224). **(B)** In the first internal cohort, the ROC curve and AUC at 1-year, 3-year, and 5-year survival were calculated. **(C)** Kaplan–Meier survival curves for the second internal cohort (n = 224). **(D)** In the second internal cohort, the ROC curve and AUC at 1-year, 3-year, and 5-year survival were calculated. Lnc, long non-coding; LUAD, lung adenocarcinoma; LUADSenLncSig, LUAD senescence lncRNA signature; ROC, receiver operating characteristic; AUC, area under the curve; OS, overall survival; TCGA, The Cancer Genome Atlas.

The TCGA-LUAD patients (n = 448) were randomly assigned to two internal validation cohorts (n = 224 in the first internal cohort and n = 224 in the second internal cohort) to determine if LUADSenLncSig is universally applicable to the OS predictive value of LUAD. The clinical characteristics of the sample are detailed in Table 1. Patients in the high-risk group had a shorter OS than subjects in the low-risk group in the first and second internal cohorts (Figures 8A,C), which was consistent with the overall TCGA-LUAD dataset results. Furthermore, the AUC of 1-year, 3-year, and 5-year survival in the first internal cohort was 0.787, 0.683, and 0.79, respectively (Figure 8B), and in the second internal cohort was 0.629, 0.679, and 0.734, respectively (Figure 8D). These results showed that LUADSenLncSig performs adequately in both internal validation cohorts, indicating the robustness of the prediction model.

**TABLE 1:** Clinical characteristics of the cancer genome atlas lung adenocarcinoma the first internal cohort and the second internal cohort (*n* = 448).

Covariates	Sub-group	Entire LUAD, n (%)	The first internal cohort, n (%)	The second internal cohort, n (%)	p Value
Age (yr)	≤65	218 (48.66%)	111 (49.55%)	107 (47.77%)	0.7767
	>65	230 (51.34%)	113 (50.45%)	117 (52.23%)	
Gender	Female	246 (54.91%)	126 (56.25%)	120 (53.57%)	0.635
	Male	202 (45.09%)	98 (43.75%)	104 (46.43%)	
Stage	Stage I	240 (53.57%)	124 (55.36%)	116 (51.79%)	0.7379
	Stage II	103 (22.99%)	49 (21.88%)	54 (24.11%)	
	Stage III	73 (16.29%)	35 (15.62%)	38 (16.96%)	
	Stage IV	24 (5.36%)	14 (6.25%)	10 (4.46%)	
	Unknow	8 (1.79%)	2 (0.89%)	6 (2.68%)	
T	T1	153 (34.15%)	79 (35.27%)	74 (33.04%)	0.3088
	T2	237 (52.9%)	110 (49.11%)	127 (56.7%)	
	T3	37 (8.26%)	22 (9.82%)	15 (6.7%)	
	T4	18 (4.02%)	11 (4.91%)	7 (3.12%)	
	Unknow	3 (0.67%)	2 (0.89%)	1 (0.45%)	
M	M0	299 (66.74%)	148 (66.07%)	151 (67.41%)	0.6652
	M1	23 (5.13%)	13 (5.8%)	10 (4.46%)	
	Unknow	126 (28.12%)	63 (28.12%)	63 (28.12%)	
N	N0	288 (64.29%)	149 (66.52%)	139 (62.05%)	0.1897
	N1	84 (18.75%)	35 (15.62%)	49 (21.88%)	
	N2	63 (14.06%)	30 (13.39%)	33 (14.73%)	
	N3	2 (0.45%)	2 (0.89%)	0 (0%)	
	Unknow	11 (2.46%)	8 (3.57%)	3 (1.34%)	

LUAD, The cancer genome atlas lung adenocarcinoma; T, Tumor; M: metastasis; N, Lymph node. The *p* value is indicated for the chi-square test and Kruskal–Wallis test among the three groups.

### Identification of biological pathways associated with LUADSenLncSig

**FIGURE 9:**
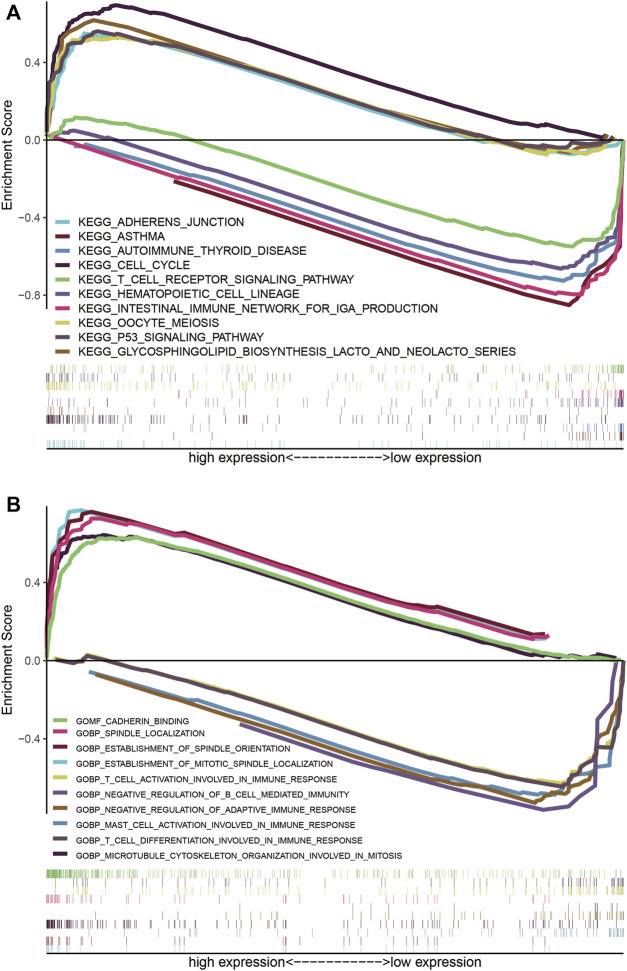
LUADSenLncSig-based GSEA of different risk groups. **(A)** Based on the GSEA results, the KEGG genes were differentially enriched for senescence-related lncRNA expression. Five KEGG items, the cell cycle, p53 signaling pathway, oocyte meiosis, glycosphingolipid biosynthesis-lacto and neolacto series, and adherens junction were enriched in the high-risk group. Asthma, intestinal immune network for IgA production, hematopoietic cell lineage, autoimmune thyroid disease, and T cell receptor signaling pathway were enriched in the low-risk group based on the NES, NOM *p*-value, and FDR q-value **(B)** Differential enrichment of genes in GO with senescence-related lncRNAs. Five GO items, spindle localization, establishment of spindle orientation, establishment of mitotic spindle localization, cadherin binding, and microtubule cytoskeleton organization involved in mitosis showed a significant differential enrichment in the high expression phenotype. The other five GO terms, negative regulation of adaptive immune response, mast cell activation involved in immune response, T cell activation involved in immune response, T cell differentiation involved in immune response, and negative regulation of B cell mediated immunity, were found significantly enriched in the low expression phenotype based on the NES, NOM *p*-value, and FDR q-value. The reference files from the (MSigDB) were c2. cp.kegg.v7.4. symbols.gmt and c5. go.v7.4 symbols. gmt (http://software.broadinstitute.org/gsea/msigdb/index.jsp). LncRNAs, long non-coding RNAs; LUAD, lung adenocarcinoma; LUADSenLncSig, LUAD senescence lncRNA signature; GSEA, Gene Set Enrichment Analysis; KEGG, Kyoto Encyclopedia of Gene and Genome; NES, normalized enrichment score; NOM *p*-value, nominal *p*-value; FDR, false detection rate; GO, Gene Ontology; MSigDB, Molecular Signatures Database.

The KEGG pathways of cell cycle (NES = 2.17, NOM *p* < 0.001, FDR q = 0.004), p53 signaling pathway (NES = 2.04, NOM *p* < 0.001, FDR q = 0.019), oocyte meiosis (NES = 2, NOM *p* < 0.001, FDR q = 0.023), glycosphingolipid biosynthesis-lacto and neolacto series (NES = 1.95, NOM *p* = 0.004, FDR q = 0.03), and adherens junction (NES = 1.95, NOM *p* = 0.004, FDR q = 0.028) were enriched in the high-risk group (Figure 9A), however, asthma (NES = −2.16, NOM *p* < 0.001, FDR q = 0.003), intestinal immune network for IgA production (NES = −2.11, NOM *p* < 0.001, FDR q = 0.004), hematopoietic cell lineage (NES = −2.1, NOM *p* < 0.001, FDR q = 0.003), autoimmune thyroid disease (NES = −2.09, NOM *p* = 0.002, FDR q = 0.003), and T cell receptor signaling pathway (NES = −2.01, NOM *p* = 0.004, FDR q = 0.009) were enriched in the low-risk group (Figure 9A). On the other hand, the GO terms enriched in LUAD patients with high-risk scores were spindle localization (NES = 2.53, NOM *p* < 0.001, FDR q < 0.001), establishment of spindle orientation (NES = 2.49, NOM *p* < 0.001, FDR q < 0.001), establishment of mitotic spindle localization (NES = 2.48, NOM *p* < 0.001, FDR q < 0.001), cadherin binding (NES = 2.46, NOM *p* < 0.001, FDR q < 0.001), and microtubule cytoskeleton organization involved in mitosis (NES = 2.35, NOM *p* < 0.001, FDR q < 0.001). Conversely, negative regulation of adaptive immune response (NES = −2.4, NOM *p* < 0.001, FDR q = 0.001), mast cell activation involved in immune response (NES = −2.26, NOM *p* < 0.001, FDR q = 0.007), T cell activation involved in immune response (NES = −2.24, NOM *p* < 0.001, FDR q = 0.007), T cell differentiation involved in immune response (NES = −2.22, NOM *p* < 0.001, FDR q = 0.008), and negative regulation of B cell-mediated immunity (NES = −2.22, NOM *p* < 0.001, FDR q = 0.006) were enriched in tumors of LUAD patients with low-risk scores (Figure 9B).

### The relationship between LUADSenLncSig and TMB

**FIGURE 10:**
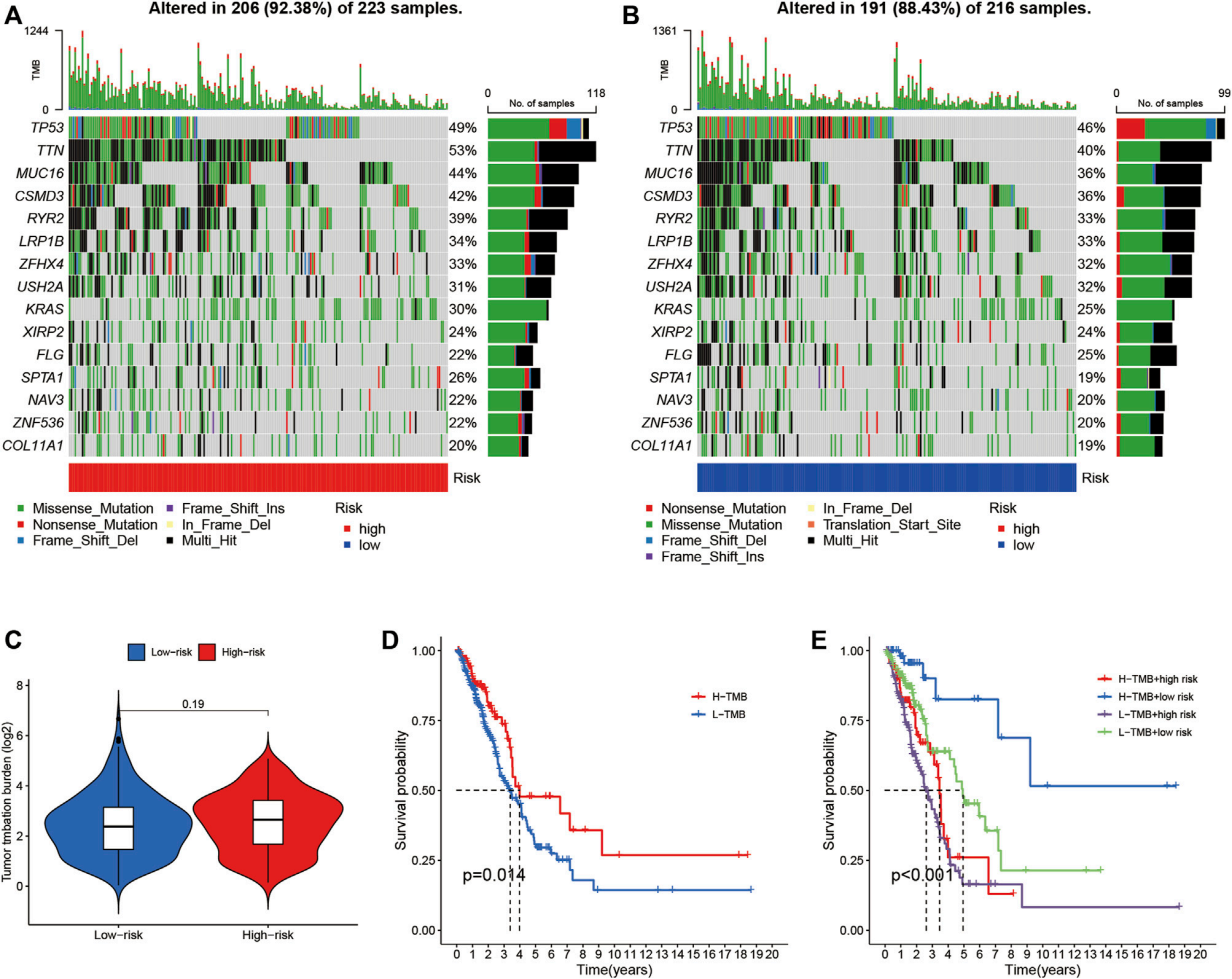
The relationship between LUADSenLncSig risk scores and somatic mutation and TMB in LUAD tissues. Waterfall plot showing the somatic mutations between high- **(A)** and low-risk **(B)** LUAD patients. **(C)** Difference of TMB between patients from the low- and high-risk score subgroups. **(D)** Kaplan–Meier curves for the high-and low-TMB groups. **(E)** Kaplan–Meier curves for patients stratified based on both TMB and risk scores. The *p*-value represents the ANOVA test between the subgroups. TMB, tumor mutation burden; LUAD, lung adenocarcinoma; lnc, long non-coding.

Somatic mutations were separately examined in 223 high-risk patients and 216 low-risk patients (Figures 10A,B). Higher mutation rates were observed in the high-risk group than in the low-risk group for TTN (53% vs. 40%), MUC16 (44% vs. 36%), CSMD3 (42% vs. 36%), and RYR2 (39% vs. 33%). In addition, although difference in TMB was not found between the high- and low-risk groups (Figure 10C), the combination of high TMB with LUADSenLncSig risk scores in the low-risk group led to better prognosis (Figures 10D,E), indicating a synergistic effect between these two indicators. In theory, a higher TMB increases tumor neoantigen production, thus, increased immune recognition and tumor cell killing are likely. In addition, an association between high TMB and ICI response rates and PFS has been found in previous studies (Sholl et al., 2020). Thus, the immune infiltration and potential immunotherapy response between the two groups were assessed.

### The profile of immune infiltration in the high- and low-risk LUAD subgroups

**FIGURE 11:**
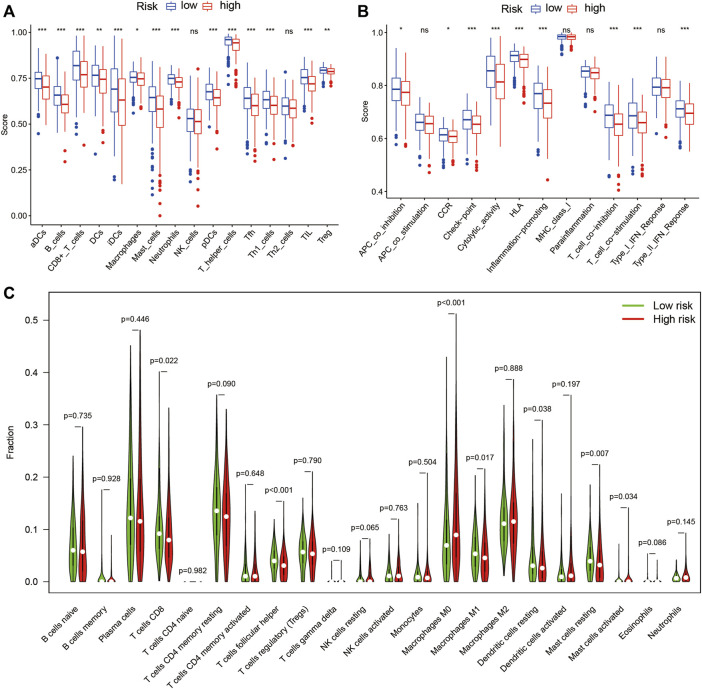
Immune cell infiltration and immune-related functions in different risk groups. The ssGSEA algorithm was used to compute the score levels of infiltration of 16 immune cells. **(A)** and 13 immune-related functions. **(B)** in the high- and low-risk groups. **(C)** The Wilcoxon rank-sum test was used to determine differences between the 22 types of immune cells between high- and low-risk groups. SsGSEA, single-sample Gene Set Enrichment Analysis; aDCs, activated dendritic cells; iDCs, immature dendritic cells; NK, natural killer; pDCs, plasmacytoid dendritic cells; Tfh, T follicular helper; Th1, T helper type 1; Th2, T helper type 2; TIL, tumor-infiltrating lymphocyte; Treg, T regulatory cell; APC, antigen-presenting cell; CCR, C-C chemokine receptor; HLA, human leukocyte antigen; MHC, major histocompatibility complex; IFN, interferon; *p < 0.05; **p < 0.01; ***p < 0.001; ns, non-significant; *p* < 0.05 indicates statistical significance.

The results of ssGSEA analysis showed the number of anti-tumor immune cells, such as CD8^+^ T cells, macrophages, and T helper cells, was significantly higher in the low-risk group than in the high-risk group (Figure 11A). Immune function scores, such as antigen-presenting cell (APC) co-inhibition, C-C chemokine receptor (CCR), checkpoint, human leukocyte antigen (HLA), T cell co-inhibition, T cell co-stimulation, and type II interferon (IFN) response, were significantly higher in the low-risk group than in the high-risk group (Figure 11B). These results indicate a stronger anti-tumor immune response in the tumor microenvironment in the low-risk group. In addition, the CIBERSORT algorithm showed the number of CD8^+^ T cells and macrophages M2 was significantly higher in the low-risk group than in the high-risk group (Figure 11C), which confirmed the ssGSEA analysis results. These differences based on the theory that immunotherapy must rely on a pre-existing immune-hot microenvironment (Mlecnik et al., 2016), provide a potential therapeutic guide for immunotherapy.

### LUADSenLncSig has a potential relationship with LUAD immunotherapy, chemotherapy, and target therapy

**FIGURE 12:**
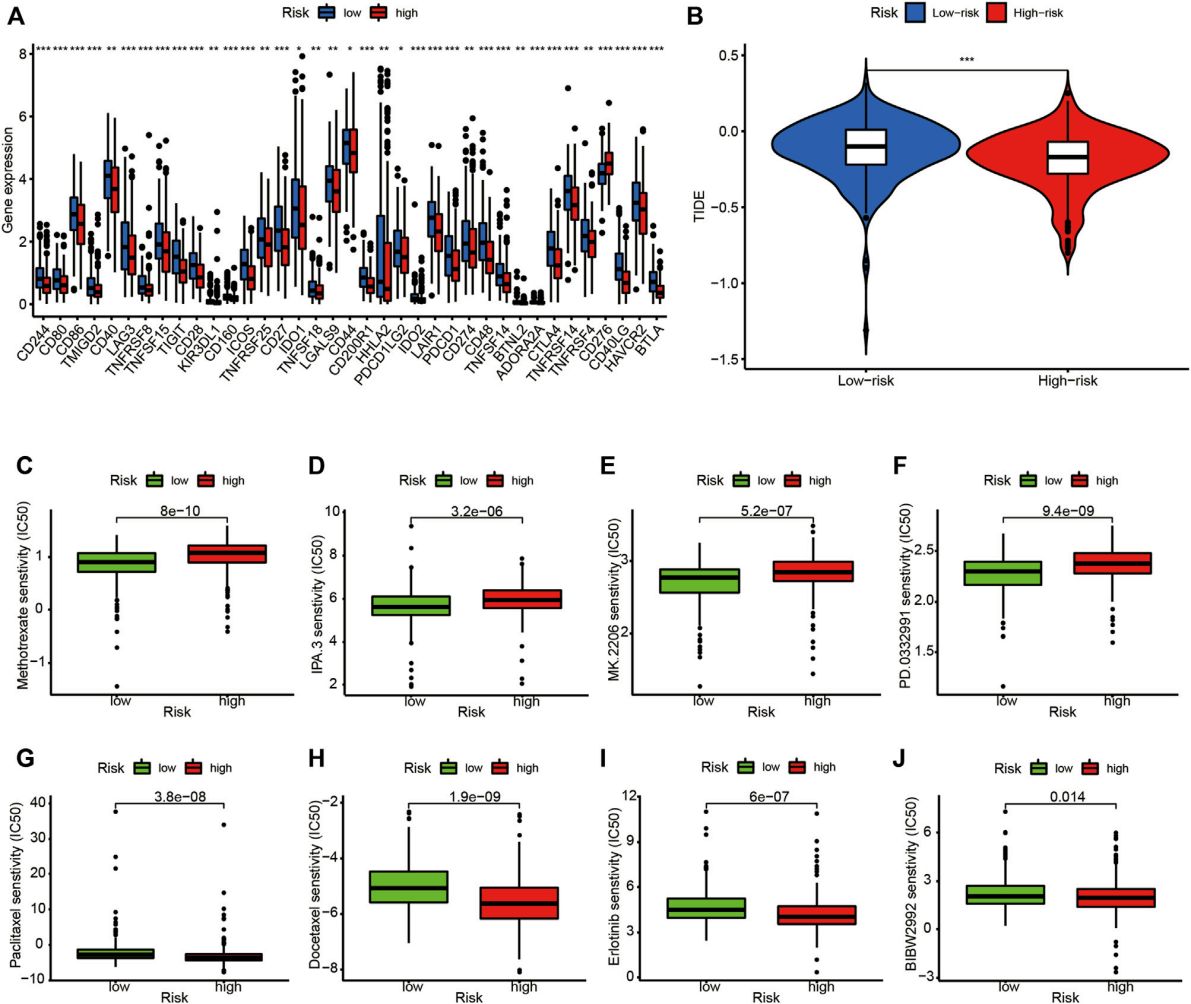
Comparison of immune checkpoints, TIDE scores, sensitivity of chemotherapy and targeted therapy drugs in the high- and low-risk groups. **(A)** Expression of 37 immune checkpoint genes differed between the high-risk and low-risk groups. Red boxes represent high-risk patients and blue boxes represent low-risk patients **(B)**. The online software TIDE prediction of the efficacy score in subgroups of LUAD patients treated with anti-PD1 or anti-CTLA4. The IC_50_ values for **(C)** methotrexate **(D)** IPA.3 **(E)** MK.2206 **(F)** PD.0332,991 (palbociclib) **(G)** paclitaxel **(H)** docetaxel **(I)** erlotinib, and **(J)** BIBW2992 (alphatinib) in the high-risk and low-risk groups. TIDE, tumor immune dysfunction and exclusion module; IC_50_, half-maximal inhibitory concentration; *p < 0.05; **p < 0.01; ***p < 0.001; ns, non-significant; *p* < 0.05 indicates statistical significance.

The expression levels of 37 immune checkpoint genes differed between the high- and low-risk groups (Figure 12A). Immunotherapy markers such as CD274 (PD-L1 protein coding gene), PDCD-1 (PD-1 protein coding gene), and CTLA-4, which are now widely used in clinical trials, were found significantly higher in the low-risk group (Figure 12A), indicating potential immunotherapeutic responses in low-risk patients. Furthermore, as shown in Figure 12B, when the online software TIDE was used to predict the efficacy of anti-PD1 or anti-CTLA4 treatment for LUAD patients, TIDE scores were significantly higher in the low-risk subgroups than in high-risk subgroups (*p* < 0.001). This finding demonstrates the LUADSenLncSig has the potential to predict immunotherapy efficacy.

Finally, the relationship between the LUADSenLncSig risk score and the efficacy of LUAD chemotherapy and target therapy was analyzed. As shown in Figures 12C–F, the traditional chemotherapy drug methotrexate (Figure 12C) as well as novel targeted therapies such as the P21-activated kinase 1 (PAK1) inhibitor IPA.3 (Figure 12D), the altered Akt inhibitor MK.2206 (Figure 12E), and the CDK4/6 inhibitor PD.0332,991 (palbociclib, Figure 12F), were among the more sensitive drugs in the high-risk group. Conversely, paclitaxel (Figure 12G) and docetaxel (Figure 12H) as well as tyrosine kinase inhibitors erlotinib (Figure 12I) and BIBW2992 (alphatinib, Figure 12J), were the more sensitive drugs in the low-risk group based on the IC_50_.

## Discussion

Lung cancer is the leading cause of cancer-related mortality worldwide. The recent FDA approval of ICIs for LUAD has changed the therapeutic landscape. However, the overall response rate of ICI is <20% (Brahmer et al., 2015) and only a subset of LUAD patients benefit from ICI treatment. Consequently, fully assessing whether patients benefit from ICI treatment is important. Biomarkers provide information for this treatment decision, however, effective biomarkers to predict efficacy in clinical applications do not currently exist (Brueckl et al., 2020). Tumor cell immunohistochemical PD-L1, plasma soluble PD-L1 (sPD-L1), TMB/blood TMB (bTMB), mismatch repair defect (dMMR)/microsatellite instability-high (MSI-H), and other biomarkers such as KRAS, STK11, KEAP1, and DNA damage response (DDR) gene variation are potential biomarkers (Doroshow et al., 2019). PD-L1 detected using tumor cell immunohistochemistry (IHC) is a standard biomarker to predict the effectiveness of ICI therapy. Pembrolizumab has been used in first-line therapy with a PD-L1 threshold of 50% in patients with advanced NSCLC (Arbour and Riely, 2019). However, PD-L1 has disadvantages of low expression and false negatives in some patients as well as spatial differences within and between tumors (Costantini et al., 2019). In the KEYNOTE-010 (Herbst et al., 2016) and KEYNOTE-042 (Mok et al., 2019) studies, patients with high TMB levels in tumor tissues who received pembrolizumab had better objective response rate (ORR), PFS, and OS than patients in the chemotherapy group (Herbst et al., 2019). However, consensus is currently lacking regarding which mutations should be applied to the calculation of TMB. In addition, because the incidence of dMMR/MSI-H in lung cancer is very low (Takamochi et al., 2017), the predictive value of immunotherapy for lung cancer must be further confirmed. Compared with a single clinical biomarker, combining multiple biomarkers into a single model can improve prediction accuracy and contribute to accurately personalized treatment planning (Guo et al., 2021). Senescent cells (regardless of cell type) have recently been identified as important functional cell types in tumor microenvironments, including LUAD (Hanahan, 2022). Pituitary tumor transforming gene 1 (PTTG1) and Holliday cross recognition protein (HJURP) are senescent cell-specific genes. PTTG1 is associated with the progression of NSCLC and a poor prognostic factor for NSCLC patients (Wang et al., 2016). HJURP is a histone H3 chaperone protein that influences mitosis cell cycle progression, DNA repair, and chromosome segregation (Wang et al., 2020b). HJURP is overexpressed in NSCLC and promotes NSCLC cell proliferation, migration, and invasion by activating the Wnt/β-catenin signal pathway (Wei et al., 2019). High HJURP expression is associated with shorter OS and disease-free survival (Wei et al., 2019). The detection of senescent cells remains controversial at present and the majority are linked to SASP expression, DNA damage, and β-galactosidase activity, none of which are specific or universal (Lee et al., 2006; van Deursen, 2014; Georgakilas et al., 2017; Kastenhuber and Lowe, 2017; Casella et al., 2019). Several lncRNAs have recently been found involved in the regulation of senescence in LUAD cells (Liu et al., 2019; Wei et al., 2019; Wan et al., 2021), however, the overall pattern of the LUAD cell senescence regulatory network remains largely unknown. Therefore, we developed a LUADSenLncSig signature that represents both senescence and prognosis of LUAD.

The LUADSenLncSig model developed in this study shows good predictive performance for the OS of TCGA-LUAD samples. Furthermore, the nomogram, which includes the LUADSenLncSig risk score, has the potential to guide clinical decisions. Notably, LUADSenLncSig can stratify LUAD patients based on immune checkpoint gene expression levels and TIDE score, which is important for the selection of patients who may benefit from immunotherapy. Among the LUADSenLncSig, LINC01116 has been shown to promote LUAD progression by affecting the p-Akt signaling pathway (Zeng et al., 2020) and epithelial mesenchymal transformation (Shang et al., 2021), and is a lncRNA associated with multiple LUAD prognostic models (Geng et al., 2021; Gong et al., 2022a; Yang et al., 2022). Although the precise mechanism of function remains unknown, SH3PXD2A-AS1 (Zhuang et al., 2022), VIM-AS1 (Zhang et al., 2021), SH3BP5-AS1 (Zhuang et al., 2022), AC092279.1 (Gong et al., 2022b), and AC026355.1 (Li et al., 2020; You et al., 2021; Zheng et al., 2021; Li and Sun, 2022) have been included in several LUAD lncRNA prognostic models. However, research on AC005838.2, AC027020.2, and LINC00996 regarding their prognostic value in cancer and contributions to senescence is lacking. Therefore, the effects of these lncRNAs on LUAD and senescence should be investigated in future studies.

The notable aspect of this study is the important relationship between cell senescence and the prognosis and microenvironment of LUAD. At present, the role of the senescent microenvironment in tumors is often ignored in preclinical studies, which are usually designed for young mice rather than old mice (Fane and Weeraratna, 2020) and may help explain why many successful preclinical responses are not reproduced after entering real clinical trials. When considering comprehensive treatment for tumors based on the role and mechanism of genes involved in the regulatory network in LUAD tissue, senescence should be included as a parameter. In a clinical retrospective study, patients >60 years of age responded better to PD-1 than patients <60 years of age (Kugel et al., 2018). Furthermore, when compared with anti-CTLA-4 and anti-PD-L1, anti-PD-1 was ineffective against melanoma in aged mice (Padrón et al., 2018). When senescent cells (expressing p16INK4a) were pharmacologically eliminated in aging mice, the incidence of spontaneous tumorigenesis and cancer-related death was reduced (Baker et al., 2016). Similarly, in the present study, the relationship between the LUADSenLncSig model derived from senescence genes and the potential efficacy of LUAD was determined. Based on LUADSenLncSig stratification, the expression of most immune checkpoints, activation of immune pathways, infiltration of anti-tumor immune cells, and TIDE score in the low-risk group were higher than in the high-risk group (Figures 11, 12), indicating that low-risk patients may benefit more from immunotherapy. In addition, compared with other studies using the TCGA database to develop lncRNA prognostic models (Bai et al., 2020; Deng et al., 2020; Zhao et al., 2020; Chen et al., 2021; Feng et al., 2021; Tang et al., 2021), our LUADSenLncSig model is very comparable. This shows that senescent cells in TME are helpful and as valuable as other biomarkers in predicting prognosis and estimating the efficacy of immunotherapy of LUAD. Interestingly, other studies have also focused on the contribution of cellular senescence-associated LncRNAs to tumor prognosis in tumors including liver (Huang et al., 2022a), colorectal (Huang et al., 2022b), and gastric (Wang et al., 2022) cancers. These studies have similar AUC values to the time-dependent ROC curves of our model and importantly illustrate the contribution of senescent cells to the tumor microenvironment.

The present study had several limitations. First, more external data should be considered to assess whether the LUADSenLncSig model fully matches the additional dataset. Second, several key data points that affect patient prognosis, such as who received second-line treatment, were lacking and could not be included in the nomogram, which may affect the model’s accuracy. Third, functional studies are needed to better understand the molecular mechanism of the lncRNA effect associated with senescence.

In conclusion, we developed a LUADSenLncSig lncRNA signature that can be used to predict LUAD prognosis. Notably, LUADSenLncSig was associated with the level of immune infiltration and the potential efficacy of tumor immunotherapy. These findings highlight the potential future direction of tumor immunotherapy with an emphasis on cellular senescence therapy. In addition, because the technology is more readily available and is becoming less expensive, bulk-sequencing is a technique that may more easily be introduced into the clinic as a standard management method. We believe that we can expand the bulk-sequencing-generated lncRNA model to the standard care of LUAD patients if sufficient external data is available, which can validate the predictive efficacy of senescence-related lncRNAs.

## Data Availability

The original contributions presented in the study are included in the article/Supplementary Material, further inquiries can be directed to the corresponding author.
